# N-glycan breakdown by bacterial CAZymes

**DOI:** 10.1042/EBC20220256

**Published:** 2023-04-18

**Authors:** Lucy I. Crouch

**Affiliations:** Institute of Microbiology and Infection, College of Medical and Dental Sciences, University of Birmingham, Birmingham B15 2TT, U.K.

**Keywords:** CAZymes, human gut microbes, N-glycan

## Abstract

The modification of proteins by N-glycans is ubiquitous to most organisms and they have multiple biological functions, including protecting the adjoining protein from degradation and facilitating communication or adhesion between cells, for example. Microbes have evolved CAZymes to deconstruct different types of N-glycans and some of these have been characterised from microbes originating from different niches, both commensals and pathogens. The specificity of these CAZymes provides clues as to how different microbes breakdown these substrates and possibly cross-feed them. Discovery of CAZymes highly specific for N-glycans also provides new tools and options for modifying glycoproteins.

## Introduction

**Figure 1:**
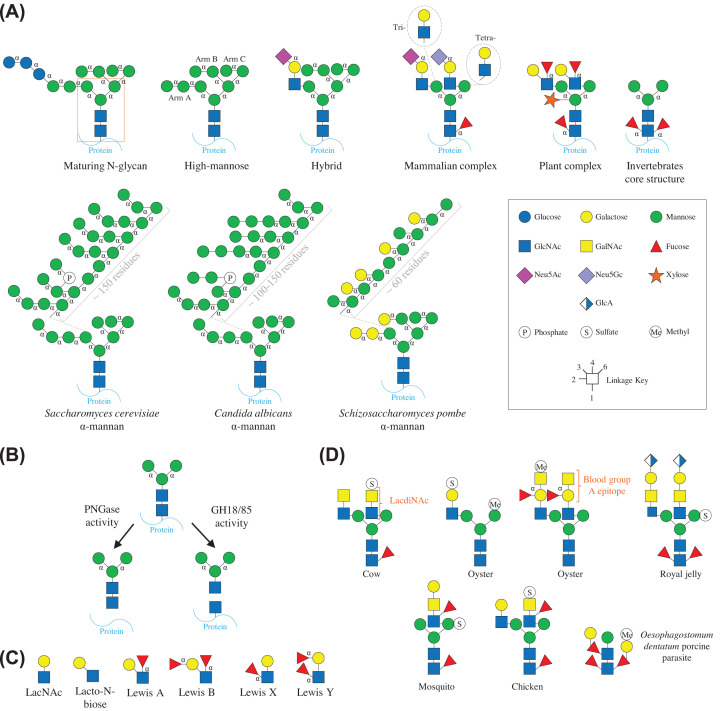
N-glycan structure guide (**A**) The structures of main types of characterised N-glycans. The core pentasaccharide of an N-glycan is outlined with an orange box, the linkages follow a linkage key and annotated with “α” for α-linkages otherwise they are β-linkages. Maturing N-glycans found in the endoplasmic reticulum have three glucose sugars on Arm A (far left). High-mannose N-glycans are composed only of α-linked mannose that have strict linkages between them as shown. Mammalian complex N-glycan is the most heterogeneous of the N-glycans, but broadly speaking their antenna is LacNAc disaccharides. For biantennary structures, these are linked to the arms through β1,2-linkages, but triantennary and tetraantennary have antenna linked through a β1,4-linkage to the α1,3 arm and then a β1,6-linkage to the α1,6 arm, respectively. The antennae are commonly capped with sialic acids, but fucose decoration is also common both on the antenna and α1,6-fucose on the core GlcNAc. There can be variations on this description, such as polyLacNAc repeats and bisecting GlcNAc linked to the core mannose via a β1,4-linkage. Hybrid N-glycans are a mix between high-mannose and mammalian complex N-glycans. Plant N-glycans have a much stricter structure, with a Lewis A epitope as antenna linked to the mannose arms via β1,2-linkages. They also have bisecting β1,2-xylose and α1,3-fucose on the core GlcNAc. Invertebrate N-glycans commonly have both α1,3- and α1,6-fucose decorating the core GlcNAc. Characterised yeast α-mannans have long α1,6-mannan extensions on the α1,3 arm, which are again decorated with more α-mannose, α-galactose, and β-mannose depending on the species [12]. (**B**) The enzyme activities of GH18, GH85, and PNGase enzymes that remove N-glycans from glycoproteins. (**C**) Useful glycan structures discussed in the review. (**D**) Examples of glycan structures adapted from [11] from a variety of organisms displaying nonclassical compositions [54–59].

N-glycans are common post-translational modifications to proteins at asparagine residues, particularly on secreted proteins, such as immunoglobulins, proteins localised to the surface of pollens, and viral proteins [1]. The composition of these glycans will vary between organisms, individuals from the same species, and also at different sites on the same protein (Figure 1). Genetics and how particular genes are regulated have a huge impact on the final glycan compositions produced in an organism. In humans, for example, fucose decorations vary considerably as some people lack the genes for producing one or two particular fucosyltransferase enzymes. In those individuals lacking these genes, this results in no Lewis A and/or Lewis B antigens, which will impact the host glycans produced [2].

Many of the CAZymes targeting N-glycans that have been characterised are those important to human health. These include enzymes acting on N-glycans produced by humans and from dietary sources derived from other animals, plants, insects, and fungi that will be nutrient sources for our gut microbiota. As a consequence, many of the bacterial enzymes analysed are from the human gut microbiota or pathogens and the presesnt review explores their activities and specificities with some comparisons from eukaryotic systems throughout. Structures of N-glycans for a wider variety of organisms have started to be explored, such as from algae, protists, worms, amoeba, sea slugs, and molluscs [3], but CAZymes specific for their breakdown are yet to be discovered.

## CAZymes that liberate N-glycans from glycopeptides

There are three families of enzymes currently described that remove N-glycans from glycoproteins – GH18, GH85, and Peptide-N4-(N-acetyl-β-glucosaminyl)asparagine amidase (PNGase) family members. GH18 and GH85 enzymes hydrolyse the GlcNAcβ1,4GlcNAc linkage in the core N-glycan pentasaccharide, whereas PNGase enzymes hydrolyse between the GlcNAc and the aparagine (Figure 1B). The characterised enzymes from the GH85 and PNGase families exclusively have activity against N-glycans. In contrast, GH18 family members have also been reported to have different specificities, such as chitinase and lysozyme activities, as well as acting on N-glycans. GH18 enzymes have only hydrolytic activities, whereas GH85 enzymes also display transglycosylation activities, although site-directed mutagenesis of catalytic residues of GH18 family members have been used to engineer this activity in [4]. In prokaryotes, these enzymes are commonly localised to the outside of the cell to remove N-glycans from glycoproteins for nutrient acquisitions. In eukaryotes, however, these enzymes usually have an alternative role in deglycosylating proteins during intracellular processing, such as during maturation of proteins in the ER and degradation for recycling [5].

### PNGases

**Figure 2:**
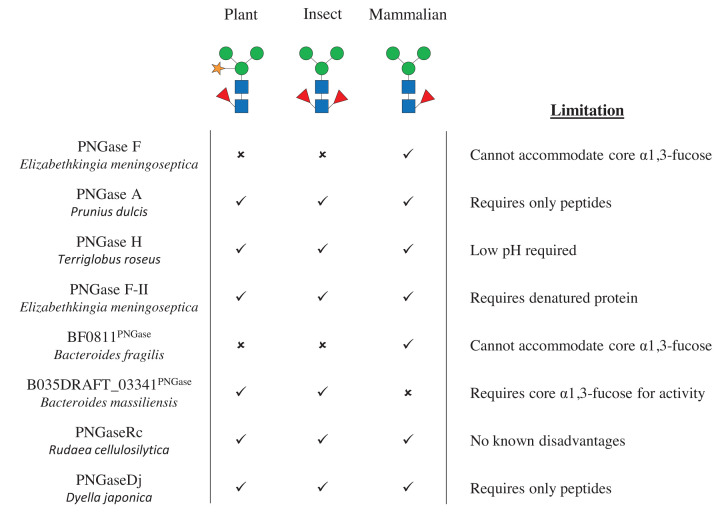
Characterised PNGase enzymes This summarises the specificities and limitations of different PNGases described so far.

The specificity of PNGases described to date is driven by the core N-glycan structure and they have broad accommodation of different types of antenna (Figure 2). PNGaseF (*Elizabethkingia meningoseptica*) is able to remove N-glycans that do not have an α1,3-fucose on the core GlcNac (characteristic of plant and insects) and works optimally when the protein is denatured [6]. PNGaseA (*Prunius dulcis*), PNGaseH (*Terriglobus roseus*), and PNGaseDj (*Dyella japonica*) can accommodate an α1,3-fucose, but can only remove the N-glycan from a peptide, is only active under low pH conditions, and has relatively low expression, respectively [7,8]. Each of these enzymes have their limitations; however, the recently discovered PNGaseRc (*Rudaea cellulosilytica*) is able to accommodate both α1,3- and α1,6-fucose when removing N-glycans from native protein at a neutral pH and retains the majority of its activity in the presence of some chemical stresses, such as urea [9]. PNGaseF-II (*E. meningoseptica*) also has specificity towards N-glycans with either α1,3- and α1,6-fucose on the core GlcNAc, but has a strong preference for denatured protein [10]. B035DRAFT_03341^PNGase^ from gut commensal *Bacteroides massiliensis* is selective for N-glycans with α1,3-fucose, and BF0811^PNGase^ from *Bacteroides fragilis* has very similar activity to PNGaseF [11].

### N-glycan endo-β-acetylglucosaminidases

There are currently two GH families that are able to remove N-glycans from glycoproteins: GH18 and GH85 families, which are much more particular about the types of N-glycan antenna they accommodate compared with PNGases.

#### Activity

The specificity of GH18 family members is highly variable, with some only targeting high-mannose [12] or complex N-glycan [13–15] structures. For removal of mammalian complex N-glycans, this family has been shown to vary in terms of their specificity in accommodating sialic acid [4,16], the requirement for core α1,6-fucose [17] or have this α1,6-fucose reduce activity [18,19], accommodating bisecting GlcNAc [16,20] or not [21], and accommodating more than two antenna [15]. There is also an example of a GH18 enzyme being specific towards a particular glycoprotein – this unique enzyme is EndoS from *Streptococcus pyogenes* that targets a specific N-glycosylation site on IgG [22].

**Figure 3:**
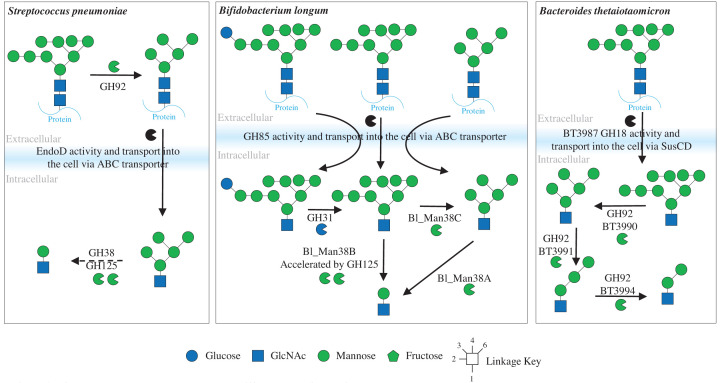
High-mannose N-glycan breakdown by different bacterial species This summarises how three different bacterial species breakdown high-mannose N-glycans. This predominantly focusses on the localisation of the different enzyme activities between these species. There is processing of the glycan prior to removal from the glycoprotein and import into the cell in the form of removing mannose from the nonreducing ends of the N-glycan for *S. pneumoniae*, but for *Bifidobacterium longum* and *B. thetaiotaomicron*, there is no processing before GH18 or GH85 activity. These models were adapted from [12,24].

**Figure 4:**
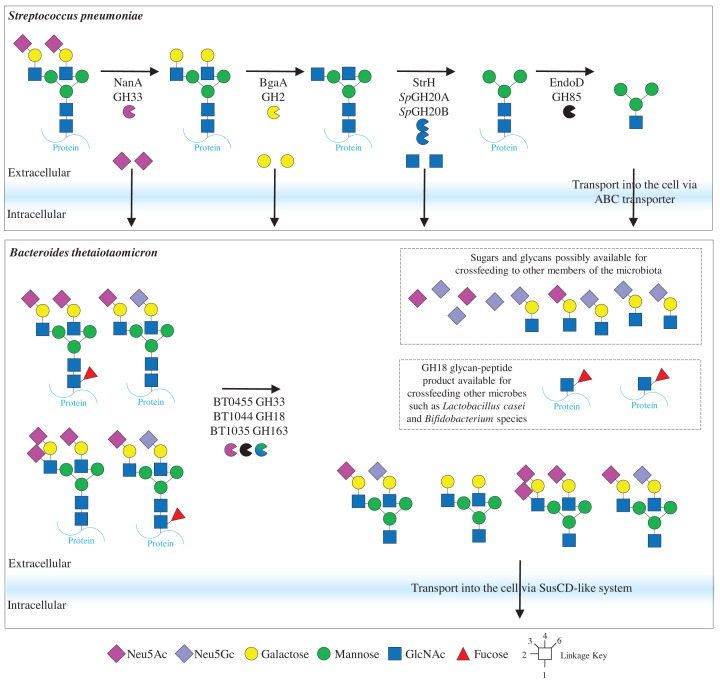
Mammalian complex N-glycan breakdown by different bacterial species This summarises how two different bacterial species breakdown mammalian complex N-glycans. This predominantly focusses the localisation of the different enzyme activities. There is extracellular processing of these N-glycans by both bacterial species, but in different ways. *S. pneumoniae* carries out sequential removal of the different sugars by exo-acting CAZymes and the sugars are all imported for use as carbon sources. *B. thetaiotaomicron* has a two endo-acting CAZymes, targeting different parts of the N-glycan and a sialidase. This also includes the fate of the GH18 glycan–peptide product being cross-fed to other microbes. These models were adapted from [16,24,40,44].

The GH85 family members characterised can also vary in their specificity. For example, EndoD from *Streptococcus pneumoniae* requires all the α1,2-mannose to be removed from high-mannose N-glycans (Man5) before hydrolysis [23]. EndoD also accommodates smaller Man3 structures, so hydrolyses complex N-glycans that have been trimmed down to the core N-glycan. These different specificities are important for understanding the biology of nutrient acquisition by different bacterial species as these endo-acting enzymes (PNGases, GH18, and GH85) will usually have to be on the outside of the cell (Figure 3). For *S. pneumoniae*, this means that the removal of nonreducing end sugars also has to happen on the surface before EndoD can remove the N-glycan from the protein [24]. In contrast, the archetypal human gut microbe *Bacteroides thetaiotaomicron* has a GH18 (BT3987) that can remove full high-mannose N-glycans, which are then imported into the cell for further degradation [12]. This species is also capable of utilising complex N-glycans, but there is some additional processing of the N-glycan on the cell surface (Figure 4) [16]. Therefore, the specificities of enzymes that remove N-glycans from the protein provides information about the biology of different bacterial species and possibly where cross-feeding of nutrients is occurring between species.

GH85 enzymes do also accommodate mammalian complex N-glycans and a recent study described three GH85 enzymes from *Tannerella* species all displaying different specificities for high-mannose, biantennary complex, and tri-/tetra-antennary complex N-glycans [6]. There has not been a GH18 or GH85 described with activity towards plant N-glycans so far.

#### Structure–function relationships

**Figure 5:**
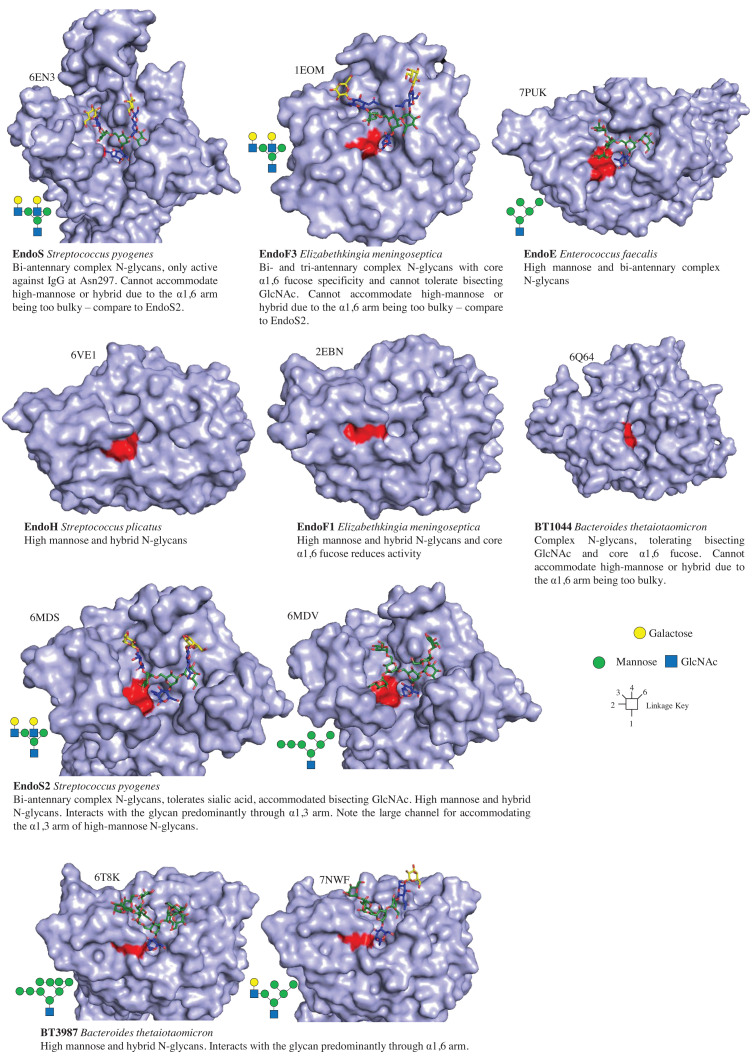
Crystal structures of GH18 family members active on N-glycans This summarises the structures available for different enzymes and where available different products bound in the active site. The products bound to the individual structures are also summarised with a glycan diagram. The different monosaccharides in the structures are colour-coded in the same way as the diagrams. The glycan always binds with α1,3 arm and the α1,6 arm in the right and left cleft, respectively. The diagnostic motif for GH18 enzymes is DXXDXDXE and the last two D and E are the two catalytic carboxylates, which are coloured red to orient the enzymes, especially those without product bound. Information is provided throughout about the origin of the enzyme, activity, and specificity.

The catalytic domains of GH18 and GH85 family members both have (β/α)_8_ barrel folds. This consists of concentric circles of the β-strands and α-helices and the active site is at the centre on the one side. The secondary structures are linked by loops, which are highly variable and drive the substrate specificity. Research into the structure–function relationship for GH18 enzymes that hydrolyse N-glycans from a number of different species has made huge progress over the last few years (Figure 5). This is largely due to solving new product–complex crystal structures but also scanning mutagenesis to understand the contribution of the different residues in the active sites [22,25,26]. A number of observations and trends are emerging from the comparison of structural data of different enzymes. In general, the majority of the interactions are with the core N-glycan pentasaccharide (Man3GlcNAc2). Furthermore, the broad-acting GH18 enzymes that can hydrolyse mammalian complex, high-mannose and hybrid N-glycans predominantly recognise the α1,3 arm of the glycan, whereas those GH18 enzymes restricted to activity against high-mannose and hybrid N-glycans predominantly recognise the α1,6 arm of the glycan [25]. Examples of these include the broad-acting EndoS2 predominantly recognising the α1,3 arm and the high-mannose/hybrid-specific BT3987 as predominantly recognising the α1,6 arm of the glycan (Figure 5). As this impacts on the way high-mannose N-glycans are recognised, the glycans adopt different conformations when bound to these different enzymes. Many of these GH18 structures have V-shaped binding sites for the antenna for the two arms to bind to. Some of these binding sites are very constricted to only allow relatively small N-glycans to bind, such as EndoE [27,28]. Other structures have larger more accommodating binding sites, such as that for the α1,6 arm in EndoS2, which accommodates both complex N-glycans and the bulkier high-mannose N-glycan antenna structures. Differences in the binding sites can also be seen for other N-glycan decorations, such as a pocket for accommodating a bisecting GlcNAc in BT1044, but not in EndoF3, reflecting differences in their specificities.

**Figure 6:**
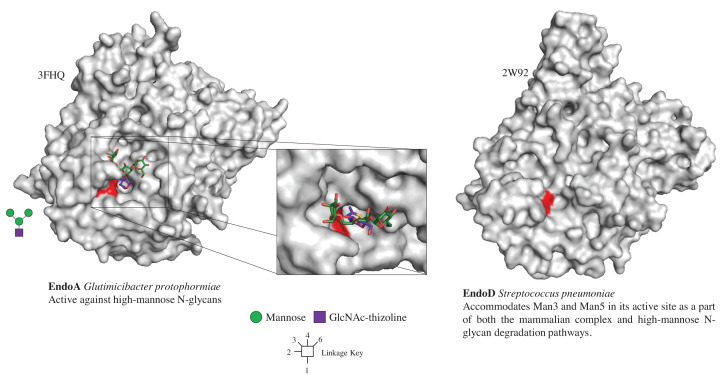
Crystal structures of GH85 family members active on N-glycans This summarises the structures available for different enzymes and where possible different products bound in the active site. The products bound to the individual structures are also summarised with a glycan diagram. The different monosaccharides in the structures are colour-coded in the same way as the diagrams. The glycan binds with α1,3 arm and the α1,6 arm in the right and left cleft, respectively. Two residues important for catalysis are an asparagine and glutamic acids, which have been coloured red for each enzyme. Information is provided throughout about the origin of the enzyme, activity, and specificity. The active site for EndoA has also been shown from above to emphasise how the protein clasps the N-glycan substrate.

There are two GH85 structures available from *S. pneumoniae* (EndoD) and *Glutamicibacter protophormiae* (EndoA) (Figure 6). The EndoD structure does have two channels for the two arms of the N-glycan akin to many of the GH18 structures. The EndoA structure has Man3GlcNAc-thiazoline bound and is unusual in having a tyrosine from one loop positioned over the exposed side of the N-glycan, specifically the central mannose and the α1,6 arm mannose. This enzyme appears to pin down the N-glycan when it is in the active site (Figure 6).

## Demannosylation of high-mannose N-glycans

The deconstruction of high-mannose N-glycans requires exo-acting α-mannosidases and those characterised so far come from the GH38, GH92, and GH125 families. The GH38 and GH92 enzymes remove mannose from the nonreducing ends and display a variety of linkage specificities, whereas the GH125 shows specificity towards α1,6-linked mannose only. For instance, three GH92 enzymes from *B. thetaiotaomicron* that are in the same operon as the GH18 BT3987 act in sequence to remove the α1,2-, α1,3-, and the first α1,6-mannose to leave Man2 (Figure 3) [12]. In terms of plant N-glycans, the human gut commensal *B. massiliensis* and the plant pathogen *Xanthomonas campestris* have both been shown to encode a GH92 with specificity towards the core α1,3-mannose in plant N-glycans, so it is able to accommodate the β1,2-xylose in its binding site [11]. A recent study of three GH38 enzymes and a GH125 enzyme from *Bifidobacterium longum*, whose genes are encoded in the same operon, shows overlapping activities towards the linkages in high-mannose N-glycans (Figure 3) [29]. Notably, it has also recently been demonstrated that a *Bifidobacterium* strain encoding the genes to breakdown high-mannose N-glycans can outcompete another strain that does not have these genes in mice, which is supportive of this substrate being important for survival in the host environment [30]. Models of the *B. thetaiotaomicron* and *B. longum* high-mannose N-glycan breakdown systems localise all these exo-acting mannosidases to inside the cell, so demannosylation happens after the whole glycan has been transported inside. In contrast, for *S. pneumoniae*, removal of the α1,2-linked mannose occurs on the outside of the cell by a GH92 to produce the Man5 substrate for the GH85 EndoD as described above (Figure 3) [24].

## Deconstruction of fungal α-mannan

The most researched fungal α-mannans are from species used in fermentation in food and beverage preparation. α-Mannans are a part of the cell wall in fungi and have huge extensions of predominantly α-linked mannose, but decorations also consist of α-galactofuranose and phosphate linking two mannose, for example (Figure 1A). Other fungi also likely produce α-mannans with additional structural variations, but this is an underexplored area.

The most detailed characterisation of the deconstruction of α-mannan was completed for brewer’s yeast *Saccharomyces cerevisiae* by the human gut microbe *B. thetaiotaomicron*, which produces endo-acting GH76 and GH99 enzymes as well as exo-acting GH38 and GH92 enzymes to do this [12]. On the cell surface, the GH99, an endo-α1,2-mannosidase, acts first to provide greater access for a GH76 to the long α1,6-mannan backbone. Together these enzymes produce α-mannan fragments and these are then imported into the cell for further deconstruction by α1,2- and α1,3-specific GH92 enzymes and then a broad-acting GH38 removes sterically restricted α1,2-mannose and mannose attached via phosphate. Phosphate is removed with phosphatases and another GH76 is then able to breakdown the α1,6-mannan backbone into shorter oligosaccharides that are substrates for a GH125. This microbe also had the capability to breakdown α-mannans from another brewing yeast, *Schizosaccharomyces pombe*, which are capped with α-galactose. A GH97 identified in the gene up-regulation data could remove this sugar. The characterisation of this system highlighted the importance of understanding how diet effects the colonic microbiota as many of the foods we eat are fermented with yeast. It was also the first demonstration of the ‘selfish’ acquisition of nutrients in the gut environment, where this species does not share α-mannan fragments with other *Bacteroides* species.

A subsequent study characterised a GH76 enzyme from a marine bacterial species of *Salegentibacter* showed endo α1,6-mannanase activity against yeast α-mannan, which suggests that fungi in aquatic environments also produce α-mannan for their cell walls [31]. The gene for this GH76 is in an operon with CAZymes from families GH92, GH2, GH125, and GH43, so it is likely this bacterium is equipped to breakdown complex fungal α-mannan structures.

## Complex N-glycan breakdown

Mammalian complex N-glycans are extremely heterogeneous in terms of the composition of the antennae and this is true even for single glycoproteins (microheterogeneity). These antenna structures share significant overlap with the nonreducing ends of O-glycans and human milk oligosaccharides (HMOs), so some CAZymes from bacteria likely have multiple targets, especially in the human gut environment. Mammalian complex N-glycan antenna are generally Lewis X epitopes and plant complex N-glycan antenna have Lewis A epitopes and are much more invariable.

### Sialic acid

Sialic acids typically cap the nonreducing ends of mammalian complex N-glycans. It also caps host O-glycan structures, so is consequently a very abundant sugar in the GI tract. Sialidase activities are carried out by GH families 33, 34, 83, and 156. GH33 enzymes are the most common family present in gut microbes and therefore the most studied in this context. The GH156 family was discovered through functional metagenomics of environmental DNA isolated from a thermal spring and homologues are predicted to be in only bacterial species [32], which have also recently be identified to be encoded in the genomes of human gut microbes [33]. Enzymes from GH34 and GH83 families are only encoded by viruses.

Sialic acids are unusual in having nine carbons and come in a number of different varieties. Humans are able to synthesise Neu5Ac (N-acetylneuraminic acid), but other animals can convert Neu5Ac to Neu5Gc (N-glycolylneuraminic acid). Incorporation of animal products into the human diet does however result in Neu5Gc being incorporated on to host glycans. A recent study showed that a Neu5Gc diet influences the composition of the microbiota, with increased numbers of Bacteroidales and Clostridiales, driven by sialidases showing preferences for different types of sialic acid [34].

As it is one of the most accessible sugars available to the microbiota from host glycans, many bacterial species have the capability to remove it for use as a nutrient source or to remove it to access other sugars and the sialic acid is then available to other microbes. For example, *Bifidobacterium bifidum* has sialidases and could support the growth of *Bifidobacterium breve*, which does not have sialidases but can grow on sialic acid, with 3-sialyllactose or mucin as a substrate [35,36]. This is also the case for the pathogen *Clostridioides difficile*, which has no sialidase but can use sialic acid as a nutrient source [37]. *Akkermansia muciniphila* is a mucin-degrading specialist in the human gut and is equipped with four sialidases – three GH33 enzymes and one that will form a new GH family. These have been recently shown to have different preferences towards α2,3-linked or α2,6-linked sialic acid. A rather elaborate method to avoid sharing this nutrient is used by *Ruminococcus gnavus*, where its sialidase produces 2,7-anhydro-Neu5Ac rather than Neu5Ac to stop other microbes accessing it and only this species has the oxidoreductase to convert this to Neu5Ac [38]. Sialic acids are also commonly decorated with acetyl groups that are removed by esterases. An esterase from *Tannerella forsythia* was shown to remove a wide variety of these different acetylations from sialic acid [39].

### Fucose

Fucose also commonly decorates complex N-glycans (Figure 1). For mammals, this is an α1,3-fucose on the antenna GlcNAc and an α1,6-fucose on the core GlcNAc. For plants, this is an α1,4-fucose on the antenna GlcNAc and an α1,3-fucose on the core GlcNAc. Invertebrates commonly have both α1,3- and α1,6-fucose on the core GlcNAc. There are other types of fucose decorations observed in invertebrates, both core and antennary, but fucosidases have not been tested against these. In terms of mammalian complex N-glycans, a GH29 from *B. thetaiotaomicron* was shown to be up-regulated in RNA-seq data in response to growth on biantennary complex N-glycans and activity was observed against antennary fucose on human breast milk colostrum IgA [16]. For the hydrolysis of α1,6-fucose, GH29 enzymes from human gut microbes *B. longum subsp. infantis* and *Lacticaseibacillus casei*, and also a GH29 from silk moth [40–42]. Interestingly, these bacterial enzymes had specificity towards the Fucα1,6GlcNAc disaccharide linked to the asparagine, whereas the silk moth enzyme was able to remove core α1,6-fucose from full complex N-glycans structures, even with sialic acid decorations. The presence of α1,6-fucose on the core GlcNAc has been shown to influence the composition of the microbiota in breast-fed infant mice. When this epitope is absent, there is an decrease in *Lactobacillus*, *Bacteroides*, and *Bifidobacterium* species alongside an increase in *Lachnospiraceae* and *Akkermansia* species [43]. The metabolism of Fucα1,6GlcNAcAsn has now been described in detail in *Lactobacillus casei* [44].

Hydrolysis of core α1,3-fucose decorations characteristic of plant and invertebrate N-glycans has also been observed in GH29 enzymes from the human gut commensal *B. massiliensis* and plant pathogen *X. campestris* [11,45]. These enzymes require the N-glycan to be significantly processed before acting, suggesting that there is a strict sequence of deconstruction by these organisms for this substrate. In terms of the plant complex N-glycan α1,4-linked antennary fucose, two GH29 enzymes were identified from *B. massiliensis* that can remove this decoration from the full plant N-glycan structure [11].

### Antenna

The antenna structures of mammalian and plant N-glycan complex N-glycans are LacNAc and Lacto-N-biose, respectively, thus usually requiring β-galactosidases and β-GlcNac’ases to deconstruct complex N-glycan antenna sequentially [40–42]. β-Galactosidases from *B. thetaiotaomicron* (GH2), *S. pneumoniae* (GH2), and *Gilkgo biloba* seeds (GH35) have been identified to hydrolyse these galactose from mammalian complex N-glycans [16,46,47]. For the removal of the final GlcNAc, a number of N-glycan-specific GH20 enzymes have been described from *S. pneumoniae* and *B. thetaiotaomicron*. SpGH20A from *S. pneumoniae* can remove GlcNAc from either N-glycan arm, but cannot remove bisecting β1,4-GlcNAc linked to the first core mannose, and SpGH20B can hydrolyse the bisecting mannose as well as the GlcNAc linked to the α1,3-mannose [48]. These two enzymes are produced as one protein and localised to the outside of the cell for processing of N-glycans on the surface (Figure 4). SpGH20C displayed broader activity towards both GlcNAc and GalNAc substrates, so likely targets other types of host glycans [49]. *B. thetaiotaomicron* has two GH20 enzymes (BT0459 and BT0506) with broad specificity for antenna GlcNAc like SpGH20A and also one (BT0456) with preference for bisecting GlcNAc and only one arm. *B. thetaiotaomicron* also produces a fourth GH20, BT0460, capable of removing any of the antenna or bisecting GlcNAc sugars [16]. The homologue for BT0459 from *B. massiliensis* is able to hydrolyse the antennary GlcNAc from plant N-glycans, thus can accommodate the bisecting β1,2-xylose [11]. This study also identified a β1,3-galactosidase, which acts before this GH20 and after the fucosidases described above.

There is one example of endo-removal of the LacNAc antenna from mammalian complex N-glycans by a GH163 from *B. thetaiotaomicron*, hydrolysing the GlcNAcβ1,2Man bond [16]. This enzyme can accommodate sialic acid linked to the galactose, polyLacNAc structures, and it can remove the GlcNAc if the galactose has already been removed. It cannot tolerate decoration of the GlcNAc by sialic acid or fucose and also cannot hydrolyse GlcNAcβ1,2Man disaccharide, which indicates a specificity towards the mammalian complex N-glycans. There was evidence for preference of one arm over the other. Furthermore, despite being predicted to be localised to the periplasm, analysis of the enzymes on the surface showed localisation of this GH163 to the cell surface. It may be that this enzyme is making mammalian complex N-glycans more easily accessible to *B. thetaiotaomicron* by breaking it down before being transported into the cell. During a growth assay, glycans being released into the media were monitored and a LacNAc sialylated with Neu5Gc remained in the media longer than other glycan fragments, suggesting this is a less preferred substrate than other parts of the N-glycan [16]. Glycans like these may be cross-fed to other species (Figure 4).

## Enzymes specific for the core N-glycan structure

The CAZymes described so far in the present review would deconstruct an N-glycan down to the Man2 structure (Manα1,6Manβ1,4GlcNAc) with either one or two GlcNAc depending on whether the N-glycan was removed by a GH18/85 or PNGase, respectively. The final α1,6-mannose has recently been shown to be removed by GH38 enzymes from *B. longum* [29]. There are examples from three different families that can breakdown the Manβ1,4GlcNAc linkage originating from both commensals and pathogens – GH2, GH5 subfamily 18, and GH130 [50–53].

## Summary

- CAZymes with different specificities towards N-glycans provide insight into how microbes process these substrates and possible cross-feeding relationships.
- Bacteria have evolved multiple CAZyme pathways to deconstruct the different types of N-glycans.
- Characterisation of CAZymes targeting host glycans will be increasingly important to our understanding of survival of microbes in and on the host.

## Abbreviations

Neu5Ac: N-acetylneuraminic acid
Neu5Gc: N-glycolylneuraminic acid
PNGase: peptide-N4-(N-acetyl-β-glucosaminyl)asparagine amidase
